# Camphor Chloral in Neuralgia

**Published:** 1874-11

**Authors:** 


Camphorated Chloral in Neuralgia.—Dr. Lennox Browne (*British Medical Journal,* March 7th) employs as a valuable topical application in neuralgia a mixture of equal parts of chloral-hydrate and gum-camphor. In every case he has found great, and often instantaneous, relief to follow its application. "Its success," he writes, "does not appear to be at all dependent on the nerve affected, it being equally efficacious in neuralgia of the sciatic as of the trigeminus. I have found it of the greatest service in neuralgia of the larynx, and in relieving spasmodic cough of a nervous or hysterical character. It is only necessary to paint the mixture lightly over the painful part, and to allow it to dry. It never blisters, though it may occasion a tingling sensation of the skin."

